# Surviving the Storm: One Young Man's Battle With Sepsis and Cardiogenic Shock

**DOI:** 10.7759/cureus.93824

**Published:** 2025-10-04

**Authors:** Samaresh C Sarkar, Ying Kristen Lau, Rhea H Kamat, Sumantra Kumar De

**Affiliations:** 1 Cardiology, London North West University Healthcare NHS Trust, London, GBR; 2 Elderly Medicine, London North West University Healthcare NHS Trust, London, GBR; 3 General Internal Medicine, London North West University Healthcare NHS Trust, London, GBR; 4 Cardiac Research, Northwick Park Hospital, London North West University Healthcare NHS Trust, London, GBR

**Keywords:** bacterial sepsis, complicated urinary tract infection, organ failure from sepsis, renal abscess complication, reversible cardiomyopathy, septic cardiomyopathy

## Abstract

Septic cardiomyopathy (SCM) is a reversible myocardial dysfunction during sepsis, marked by impaired ventricular contractility and reduced ejection fraction without primary ischaemic injury. It significantly contributes to septic shock morbidity and mortality. We report a healthy young male who developed acute left ventricular systolic dysfunction after severe urosepsis from *Escherichia coli* bacteremia. The initial assessment revealed haemodynamic instability, lactic acidosis, and a left ventricular ejection fraction (LVEF) of less than 10%. Despite fluids and vasopressors, he progressed to cardiogenic shock requiring multiple inotropes and extracorporeal membrane oxygenation (ECMO) consideration. Serial imaging revealed cardiac recovery with LVEF rising to 65% and resolving myocardial inflammation. The infection, complicated pyelonephritis with a renal abscess, was managed conservatively with prolonged antimicrobials. SCM's pathophysiology involves inflammatory mediators, endotoxins, and mitochondrial dysfunction, causing transient systolic and diastolic impairment. Early recognition via bedside ultrasound and haemodynamic optimisation are crucial for recovery. This case highlights SCM's reversibility and the need for further research into targeted therapies.

## Introduction

Septic cardiomyopathy is a complex yet potentially reversible form of cardiac dysfunction that independently accounts for the high mortality rates associated with severe sepsis and septic shock. Cardiac depression is marked by acute myocardial depression, reduction in contractility, and dilatation in the ventricle, even with elevated blood flow, causing non-response to fluid therapy [1]. Although sepsis-related myocardial depression has long been acknowledged, there is still no consensual definition. A better understanding of the specific pathophysiology of septic cardiomyopathy is necessary to develop therapies that are sufficiently potent to modulate its detrimental effects and to improve patient outcomes [2].

The syndrome is triggered by myocardial injury, systemic inflammation, cell injury, and dysfunction, all of which occur as a result of the body's response to infection and the subsequent release of powerful inflammatory mediators that have a direct action on the heart muscle [3]. According to large cohort studies and meta-analyses utilising echocardiographic criteria, we know that the prevalence of septic cardiomyopathy in sepsis patients varies from approximately 13.8% to 20%. A systematic review and meta-analysis published in 2023 reported a 20% pooled prevalence in adult sepsis patients, while a single-centre retrospective cohort study reported an incidence of 13.8% [4]. Recorded in-hospital mortality in septic cardiomyopathy shows a wide variation but commonly ranges between 20% and 50% with heterogeneity in definitions and the population studied [5].

We report a case of a young adult who developed LV dysfunction with inotropic support needed following severe urosepsis. It further emphasises the relationship between systemic infection and cardiac dysfunction and draws attention to the reasons behind and management of septic cardiomyopathy. The patient's LV function slowly improved with treatment, and inotropic agents were successfully weaned off. This is an example of reversible septic cardiomyopathy and highlights the importance of early recognition and aggressive treatment for recovery. However, this reversibility is sometimes dependent on adequate and timely treatment, as well as the absence of other comorbid cardiac diseases [6]. Therefore, understanding the exact mechanisms behind septic cardiomyopathy is essential for developing effective treatments that can mitigate its negative effects and improve patient outcomes [2].

## Case presentation

A male in his 20s with no significant past medical history presented to the emergency department in a district hospital with a day's history of feeling unwell with fever, rigours, lethargy, and dysuria. He was started on intravenous antibiotics for a urinary tract infection, with his urine dip being positive. Subsequently, a catheter urine sample was sent, which, although it did not show any bacterial growth, revealed a raised white cell count. Unfortunately, he deteriorated rapidly within the next hour, showing signs of sepsis, and was moved to the resuscitation area. He appeared pale and drowsy, with a tender abdomen predominantly in the hypochondrium, left flank, and left iliac fossa. Fluid resuscitation was initiated immediately, resulting in an initial rise in blood pressure; however, this subsequently dropped to 68/45 and at one point even became unrecordable. This drastic decline in the clinical status of an otherwise fit and well patient prompted an urgent focused transthoracic echo (TTE), which revealed severely impaired left ventricular systolic function with an ejection fraction (EF) of less than 10% with global hypokinesis. This rapid deterioration, therefore, also necessitated the involvement of the intensive care team, requiring escalation to vasopressors and inotropic support.

**Figure 1:**
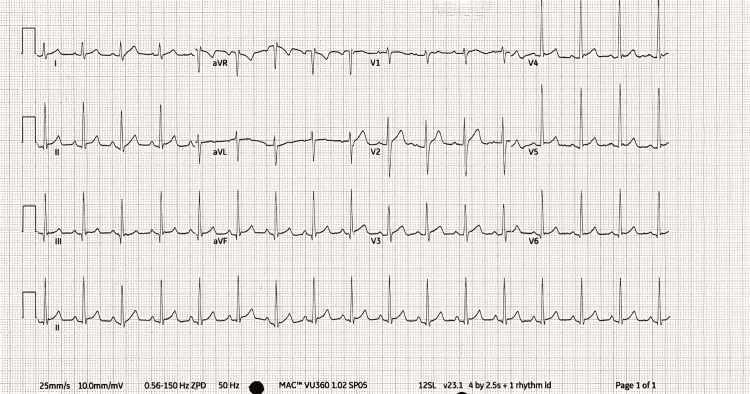
12-lead ECG on Day 0 showing sinus tachycardia.

Following initial stabilisation, a comprehensive history was taken to assess for contributing factors to his clinical presentation. There was no history of substance abuse, barring the consumption of protein supplements for bodybuilding. He had a single female partner in the preceding six months, with no signs or symptoms suggestive of sexually transmitted infection, and consequently had not undergone any screening. In terms of investigations, blood tests on admission revealed an elevated C-reactive protein, suggesting inflammation; however, both his white cell count and neutrophil count were normal. Renal function tests were acutely deranged with elevated urea and creatinine levels (Table 1). Blood gas analysis at initial presentation suggested metabolic acidosis, with a decreased pH of 7.31 and a raised lactate level of 4.4 mmol/L. However, despite initial treatment, there was further progression of tissue hypoperfusion, as evidenced by a worsening pH of 7.24 and a lactate level of 7.3 mmol/L (Table 2). A 12-lead ECG showed sinus tachycardia (Figure 1).

**Table 1:** Biochemical investigations since presentation to discharge ALT: alanine aminotransferase; ALP: alkaline phosphatase; CRP: C-reactive protein

Investigations	Range	18/03/2025	22/03/2025	26/03/2025	29/03/2025	04/04/2025
Haemoglobin	130–170 g/L	137	106	109	90	97
White Blood Cell	3.0–10.0 × 10^9^/L	5.7	15.7	19.9	13.5	6.8
Neutrophils	2.0–7.5 × 10^9^/L	5	11.2	15.9	10.5	4.2
Platelet	150–400 × 10^9^/L	150	142	415	579	622
Potassium	3.5–5.3 mmol/L	4.6	4.4	4.8	4.6	4.3
Magnesium	0.70–1.00 mmol/L	0.55	1.06	0.87	0.94	-
Sodium	133–146 mmol/L	130	137	136	137	139
Adj Calcium	2.20–2.60 mmol/L	2.3	2.32	2.44	2.58	2.52
Urea	2.5–7.8 mmol/L	8.6	6.2	4.9	6.1	6.6
Creatinine	59–104 µmol/L	175	159	143	151	121
Total Bilirubin	0–21 µmol/L	15	5	8	10	6
ALT	10–50 IU/L	43	56	81	32	46
ALP	30–130 IU/L	108	170	113	85	91
Albumin	35–50 g/L	43	31	35	39	41
CRP	0.0–5.0 mg/L	94.7	76.6	74	89.8	62.5

**Table 2:** Blood gas analysis results

Parameters	Normal Values	18/03/2025	18/03/2025	26/03/2025
		Time: 11:28	Time: 13:42	Time: 06:20
pH	7.35–7.45	7.314	7.236	7.416
Lactate	0.5–2.2 mmol/L	4.4	7.3	1.2
Base excess level	-2.7–2.5 mmol/L	2.3	-3.3	2.7
Bicarbonate level	22–29 mmol/L	30.1	25.1	28

**Figure 2:**
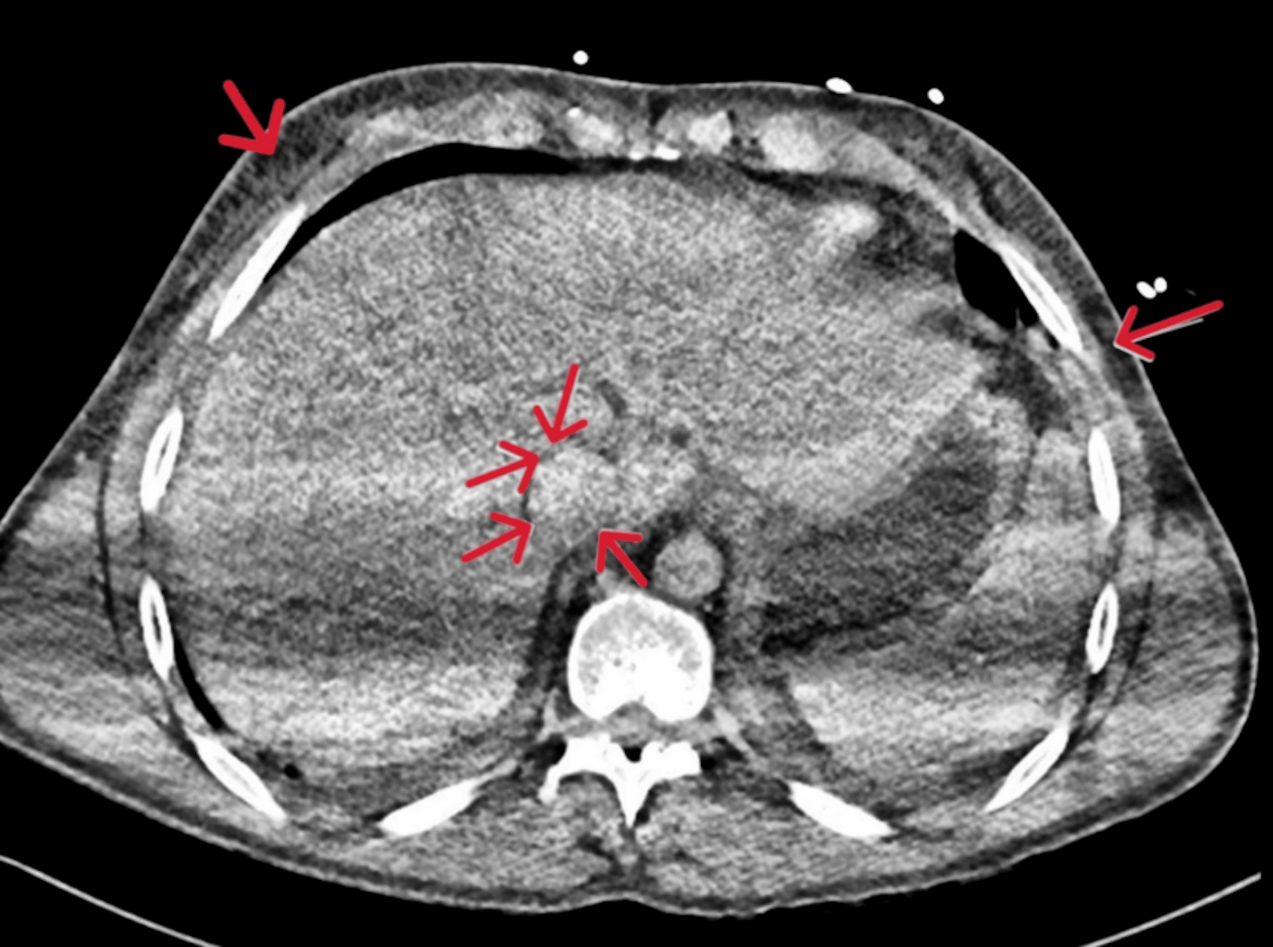
CT abdomen Day 0 transverse section showing diffusely altered attenuation of the liver with prominent hepatic veins and inferior vena cava, suggesting hepatic venous congestion. Generalised oedema of the intra-abdominal and subcutaneous fat. Normal appearance of other solid intra-abdominal organs. No hydronephrosis. Red arrows indicate prominent hepatic veins, the inferior vena cava, and generalised oedema of the subcutaneous fat.

**Figure 3:**
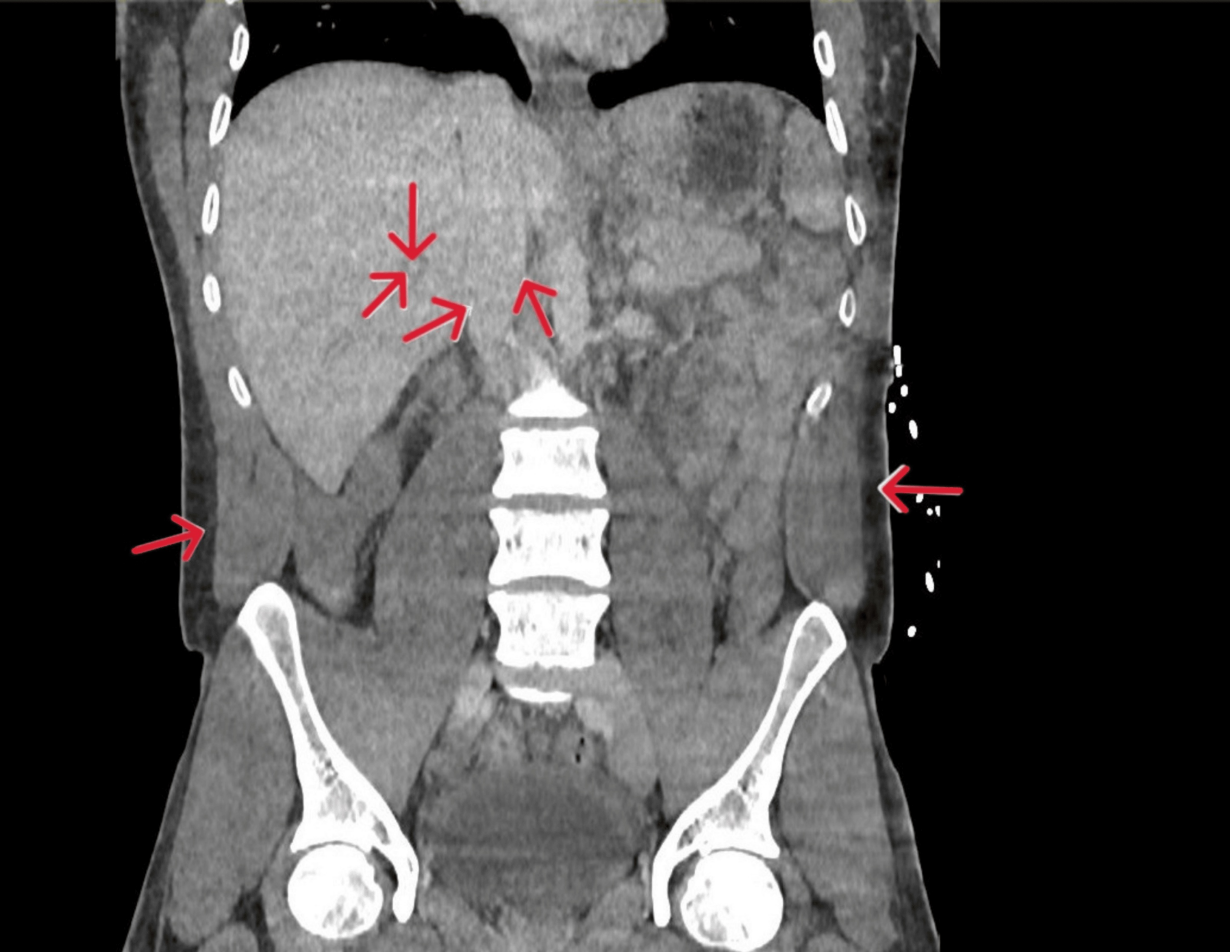
CT abdomen Day 0 coronal section showing prominent hepatic veins and inferior vena cava, suggesting hepatic venous congestion. Generalised oedema of the intra-abdominal and subcutaneous fat. Red arrows indicate prominent hepatic veins, the inferior vena cava, and generalised oedema of the subcutaneous fat.

A computed tomography (CT) scan of his chest, abdomen, and pelvis was carried out to investigate the rising lactate and inflammatory markers. This demonstrated fluid overload with no signs of bowel ischaemia (Figures 2, 3).

**Figure 4:**
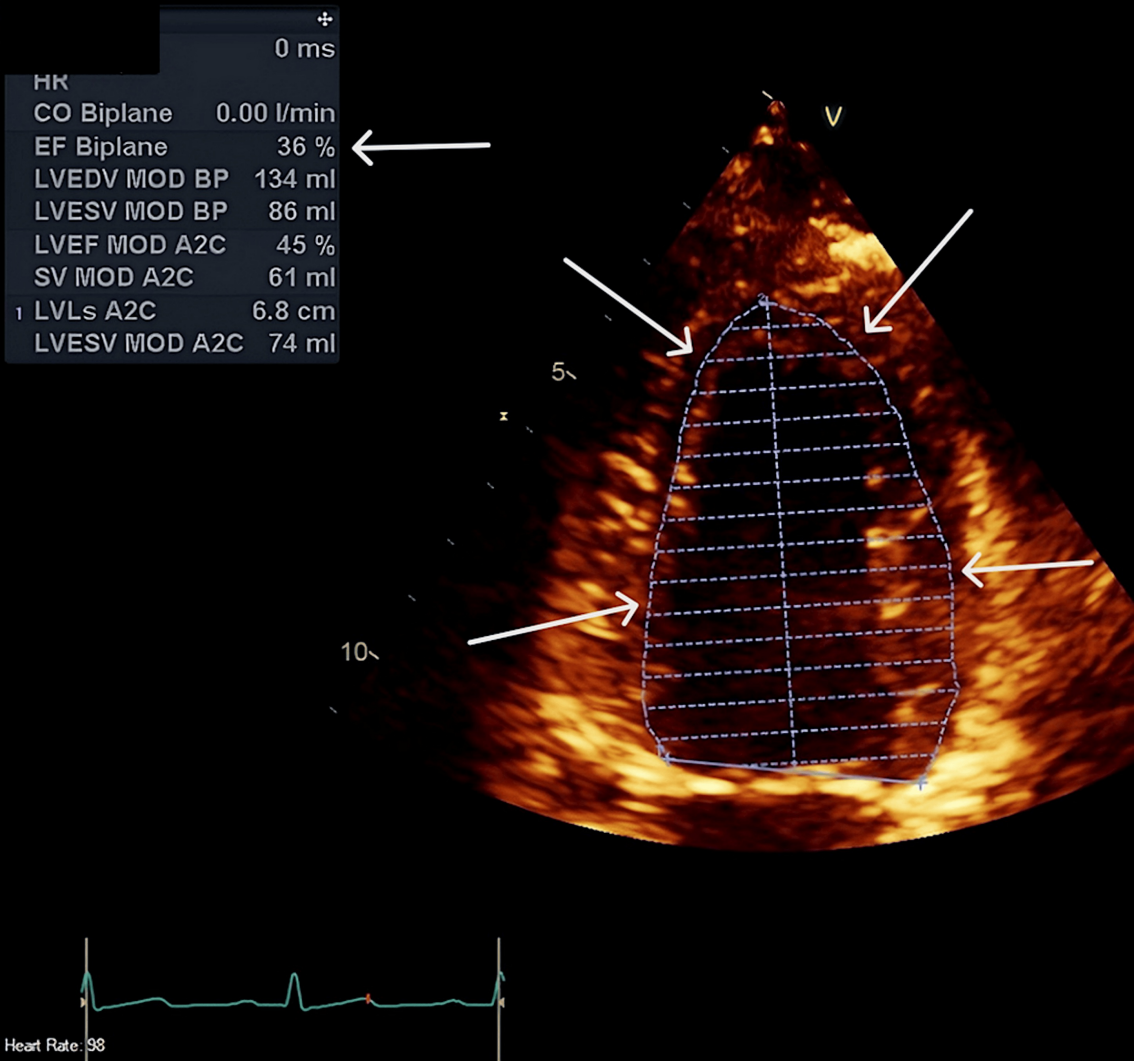
2D TTE Day 0 showing normal left ventricular cavity size with severe LV systolic dysfunction by Simpson's biplane LVEF 36%. Arrows delineating the left ventricular internal borders used to calculate the ejection fraction of 36% using Simpson's biplane. EF: ejection fraction by Simpson's biplane method; TTE: transthoracic echo

Formal 2D TTE confirmed severely impaired cardiac function with EF 36%. At this point, it was becoming evident that the patient was showing signs of early septic cardiomyopathy, comprised of severe systemic illness complicated by significant cardiac dysfunction (Figure 4).

Given the complexities involved, coupled with the lack of adequate response to initial treatments for his cardiogenic shock, he had to be transferred to a specialised centre for consideration for extracorporeal membrane oxygenation (ECMO). While being in intensive care, he continued receiving intravenous antibiotics and vasopressor and inotropic support guided by invasive arterial blood pressure and central venous pressure monitoring. The fascinating presentation and slow clinical progress prompted the consideration to investigate his cardiac impairment with a CT; however, this had to be shelved due to persistent tachycardia. Nonetheless, a repeat CT abdomen was arranged six hours post his previous, which showed new evidence of left-sided pyelonephritis. Procalcitonin levels came back elevated, suggesting an ongoing bacterial infection; this was confirmed by his blood cultures growing *Escherichia coli*. This guided further tailoring of antibiotic therapy.

**Figure 5:**
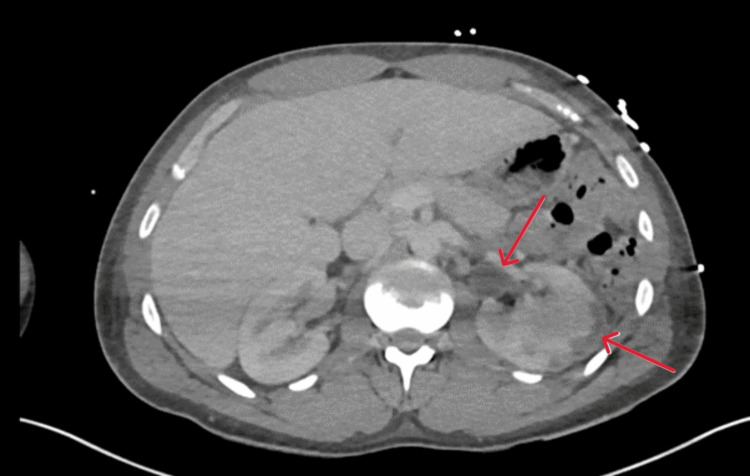
A CT abdomen on Day 5 showed worsening appearances of the left-sided severe pyelonephritis and lobar nephronia with a subcapsular collection measuring up to 1.2 cm in depth. There is mild hydronephrosis and hydroureter with an enhancing lumen, consistent with ongoing inflammation. The red arrows show left-sided severe pyelonephritis and lobar nephronia.

In the following days, he was monitored closely in the intensive care unit and was successfully weaned off vasopressor and inotropic support. After being in the ICU for nearly 90 hours, he was finally stable enough to be stepped down. Unfortunately, if one felt that the storm was over, it was not. Upon repatriation back to the district hospital, he continued to experience persistent febrile episodes. This prompted further radiological evidence in the form of an abdominal CT scan five days after his previous one, which showed worsening appearances of the previous pyelonephritis, along with lobar nephronia and a subcapsular collection (Figure 5).

**Figure 6:**
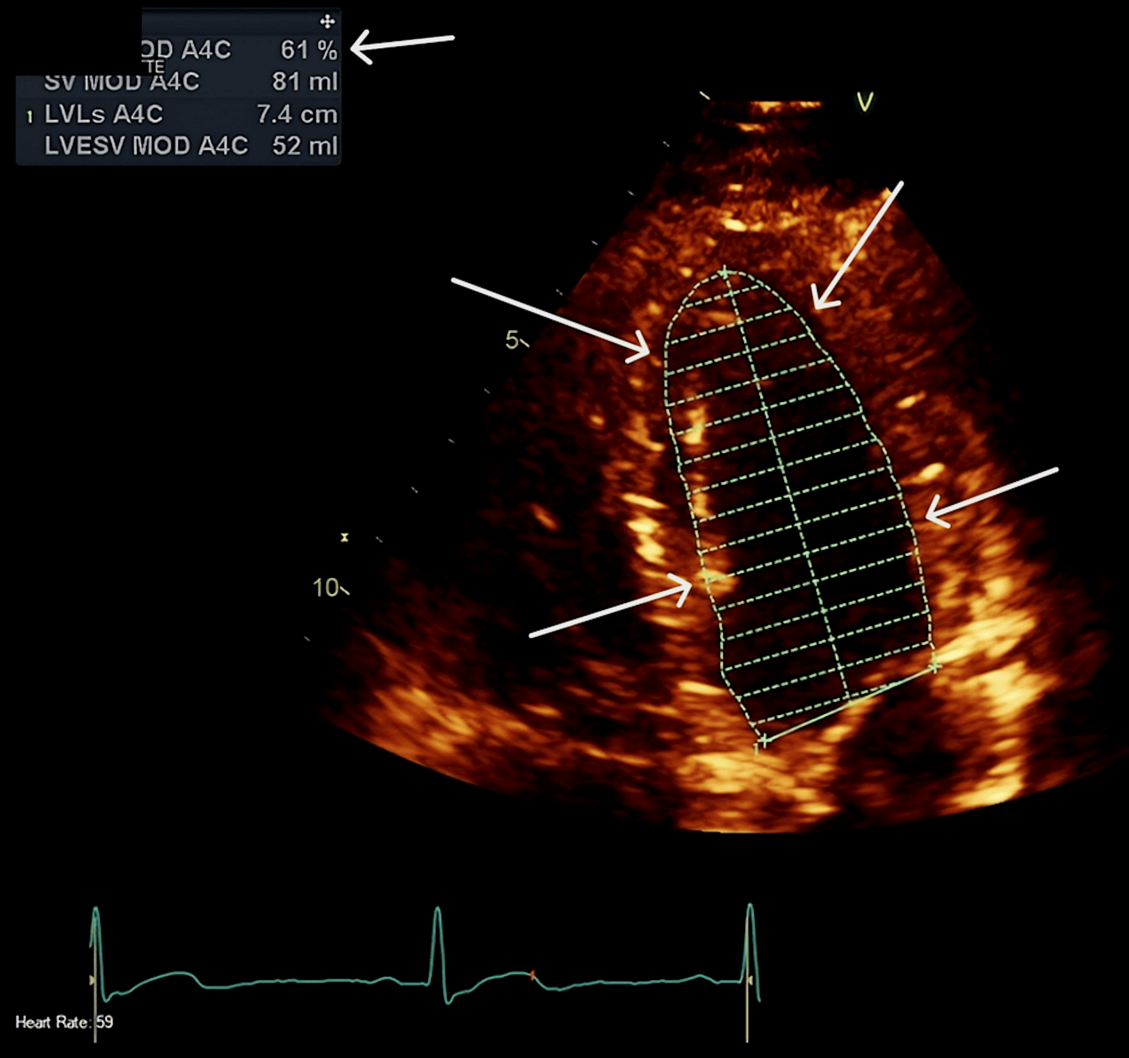
2D TTE Day 10 showing improvement of left ventricular ejection fraction to 61%. Arrows delineating the left ventricular internal borders used to calculate the ejection fraction of 61% by Simpson's biplane. TTE: transthoracic echo

**Figure 7:**
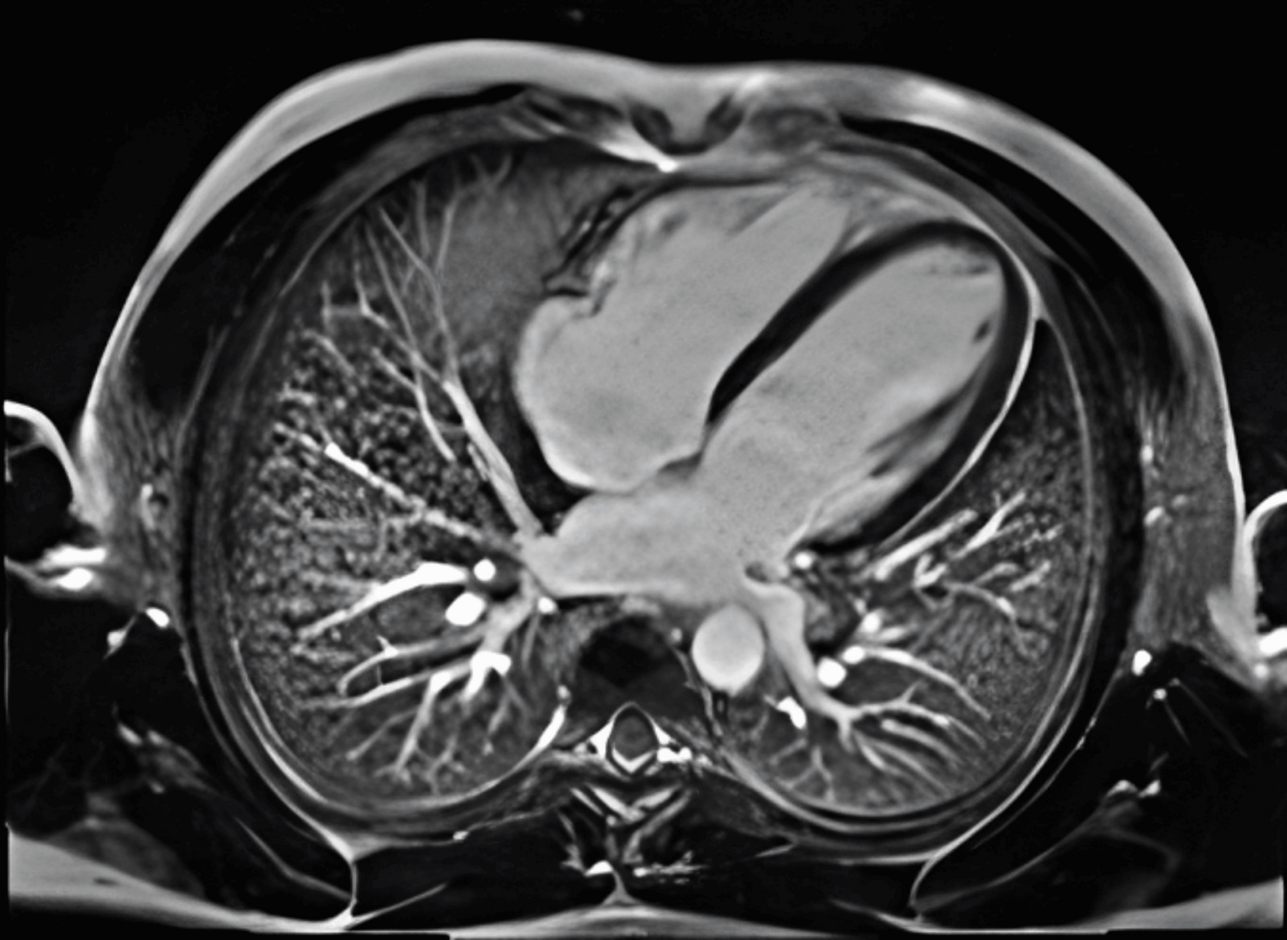
CMR Day 11 showing normal biventricular volumes and systolic function with no late gadolinium enhancement. CMR: cardiac magnetic resonance imaging

**Figure 8:**
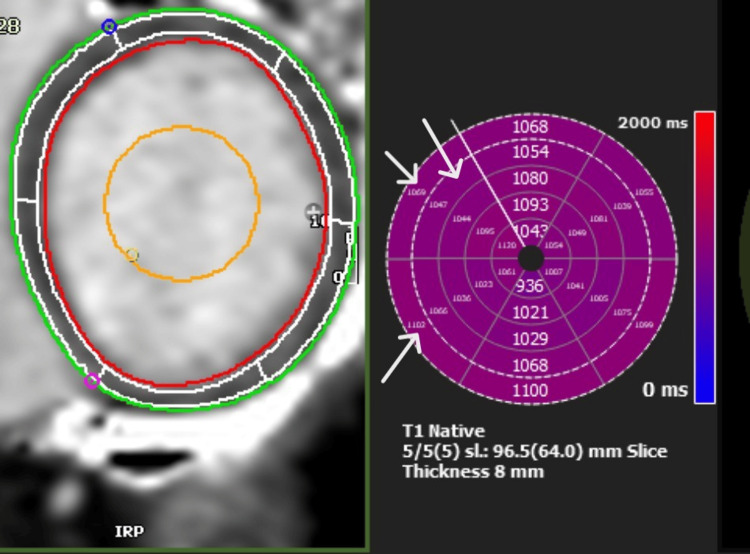
CMR Day 11: T1 Myomap sequence Global T1 is mildly elevated at 1047 ms, with a regional elevation in the basal septum from 1069 to 1107 ms. There is no evidence of oedema in these segments. Findings consistent with a resolving 'septic cardiomyopathy'. Arrows indicate regional elevation in the basal septum. CMR: cardiac magnetic resonance imaging

A multidisciplinary team approach, including cardiology, urology, interventional radiology, and microbiology, was essential. A conservative approach was opted for, with a further long course of antibiotic therapy due to the nature of the collection. In the meantime, serial TTEs were conducted, demonstrating the recovery of previously impaired cardiac function, which was also supported by cardiac magnetic resonance imaging (CMR) (Figures 6, 7). The CMR also demonstrated inflammation in the basal septum and inferior and inferolateral segments with no oedema, a finding consistent with septic cardiomyopathy. Cardiac MRI with standard T1 & T2 Myomap sequences demonstrated a global T1 elevation at 1047 ms (normal upper limit: 1030 ms for a 1.5T scanner), with regional elevation in the basal septum from 1069 ms to 1107 ms (Figure 8).

**Figure 9:**
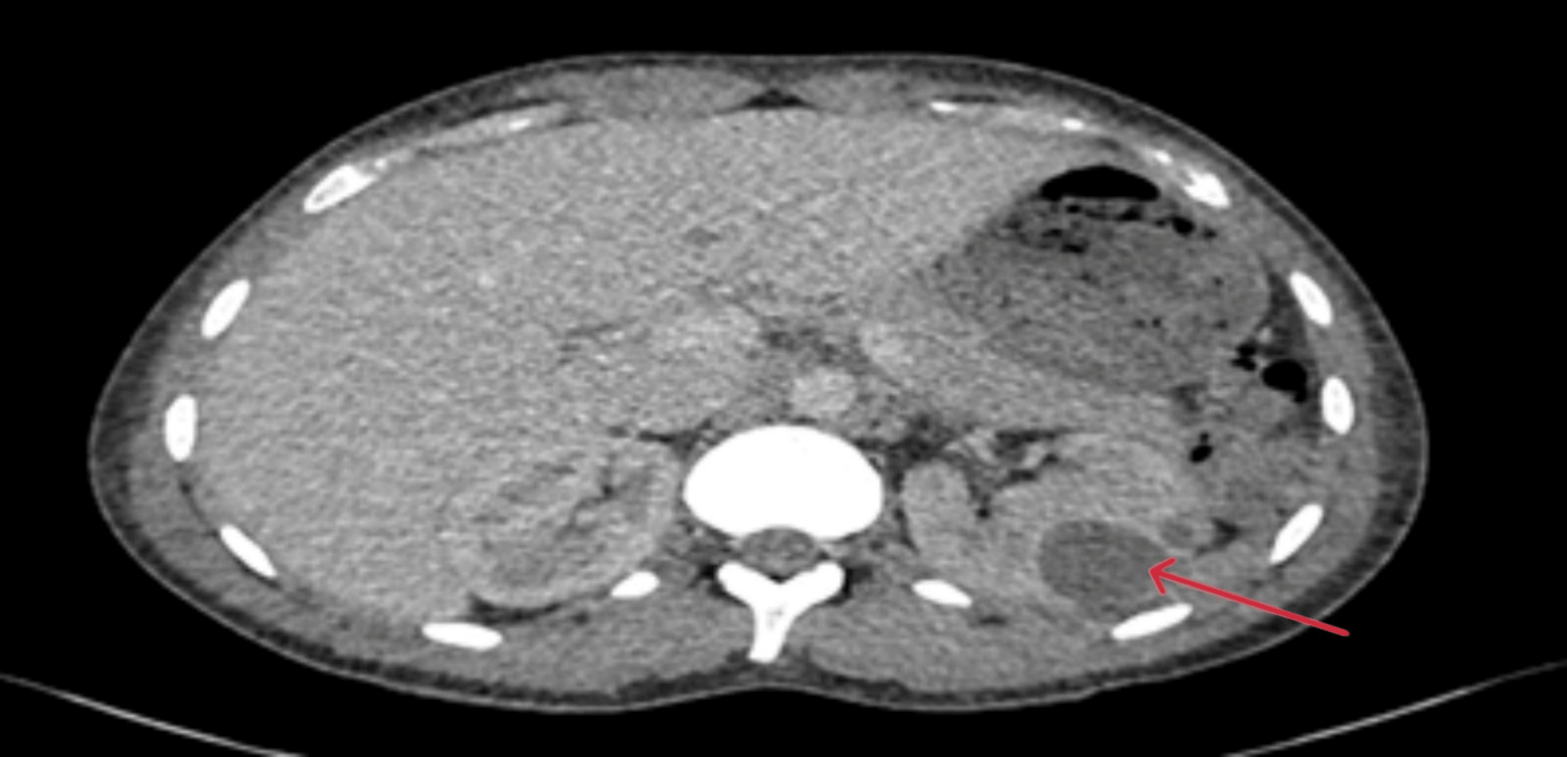
A CT abdomen on Day 12 demonstrated the interval development of a 3.5 cm renal abscess in the left interpolar region. There also remains a slightly loculated left subcapsular collection, which measures up to 1.4 cm in depth. No hydroureteronephrosis bilaterally. The red arrow indicates a renal abscess in the left interpolar region.

The following week, the patient experienced additional episodic febrile outbreaks; however, he was showing biochemical improvement. Given the turn of events, it became prudent to reevaluate whether our strategy was yielding fruitful results. Consequently, the patient underwent another CT scan of the abdomen, which revealed a 3.5 cm renal abscess, prompting us to explore options of aspiration rather than drainage as the size remained small (Figure 9).

**Figure 10:**
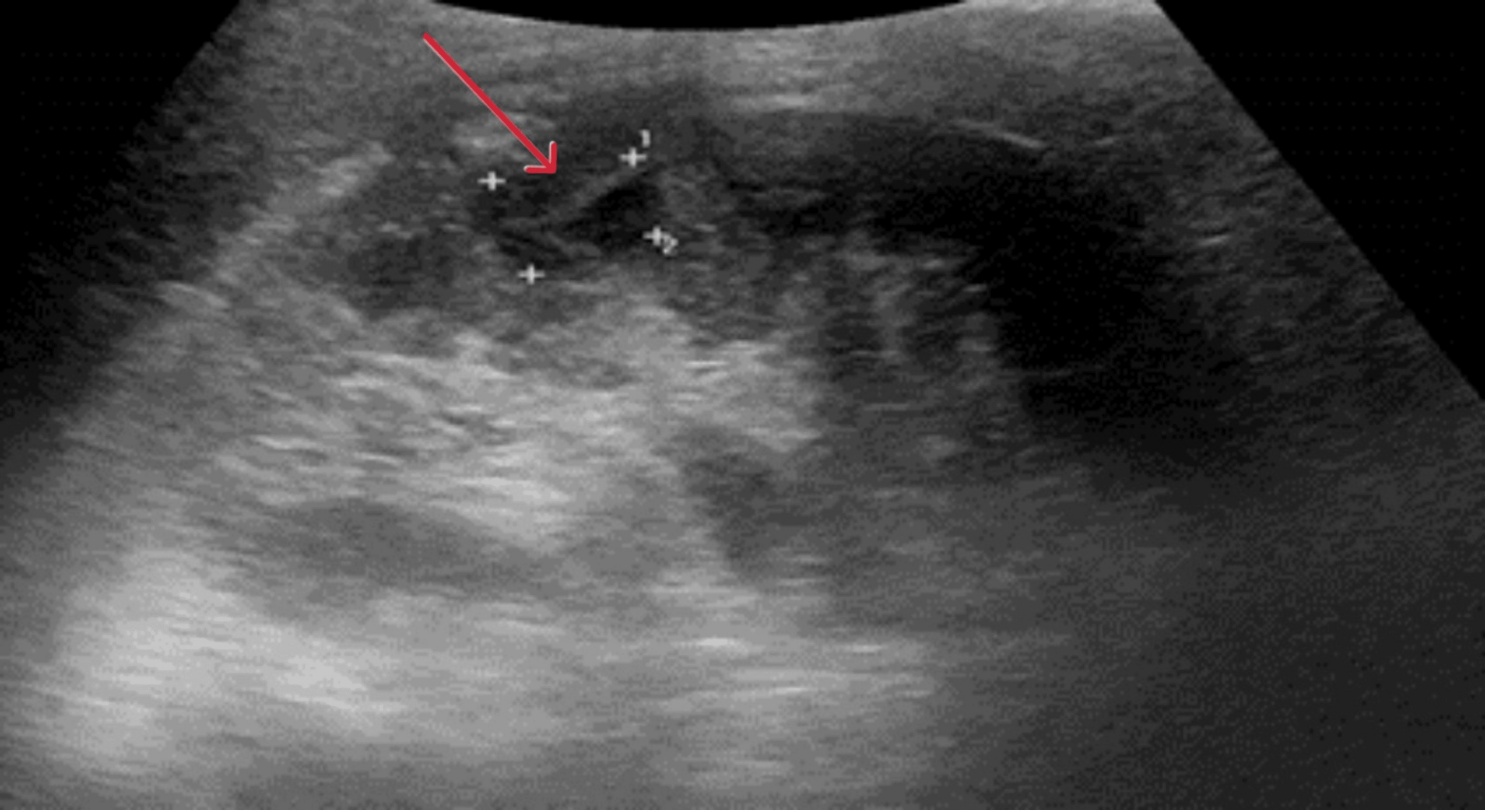
US KUB Day 46 showing the left renal abscess reduced in size, measuring 20 x 16 x 14 mm in size (tr x p x depth). Red arrow showing the presence of a left renal abscess. US: ultrasound; KUB: kidney, ureter, and bladder

**Figure 11:**
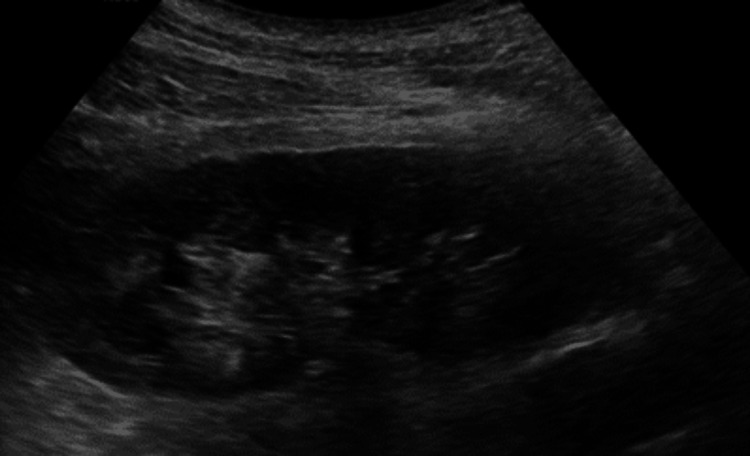
US KUB Day 134 showing complete resolution of the abscess in the left kidney. US: ultrasound; KUB: kidney, ureter, and bladder

However, this approach was not necessary because the patient stopped experiencing further pyrexia, and his biochemical markers showed significant improvement. After acute hospitalisation for nearly three weeks, he was finally discharged home with four weeks of oral antibiotics and urology follow-up. Subsequently, he attended follow-up appointments for both urology and infectious diseases and then returned to his usual routine. An ultrasound of the kidneys showed persistence of the renal abscess, albeit smaller, even four weeks after discharge (Figure 10), and it had resolved completely on the ultrasound performed after another 13 weeks (Figure 11).

## Discussion

Despite diligent investigation, the exact pathophysiological mechanisms behind septic cardiomyopathy are still incompletely understood, thereby representing a major obstacle to therapeutic progress. Cardiac dysfunction during sepsis is associated with cardiomyocyte molecular and cellular events. It is accepted that septic cardiomyopathy is characterised by left ventricular systolic dysfunction (LVSD), left ventricular diastolic dysfunction (LVDD), and right ventricular dysfunction, which contribute to the haemodynamic failure in the presence of a hyperdynamic state. Compromised ventricular response to fluid therapy and, in some cases, ventricular dilation can, in turn, further complicate the ability to maintain sufficient tissue perfusion. This hyperdynamic circulatory state, frequently observed in septic shock, exhibits some features of increased cardiac output, paradoxically occurring in the presence of myocardial depression with decreased EF and biventricular dilation [7].

The other differential that one would consider in this scenario is the possibility of myopericarditis. Septic cardiomyopathy is characterised by reversible global ventricular dysfunction, with imaging abnormalities typically resolving within 7-10 days after sepsis control without leaving any long-term cardiac sequelae, as definitively demonstrated in our case. Myopericarditis, in contrast, may show persistent imaging abnormalities, especially late gadolinium enhancement on cardiac MRI, which is robustly associated with an increased risk of adverse long-term outcomes, including heart failure, arrhythmias, and progression to dilated cardiomyopathy [8].

Diagnostic challenges of septic cardiomyopathy add to the complexity in the management of patients since there is no widely accepted definition, and there is a dearth of large epidemiological studies on its true prevalence [9]. In the present case, the patient's clinical course supports the concept that cardiac failure can present early in sepsis, reflected by his initial decreased left ventricular function despite presenting with a hyperdynamic state. This early subclinical phase of cardiac involvement, frequently hidden behind compensatory mechanisms, is highly predictive of a bad prognosis when timely diagnosed and treated. The setting in which the heart is unable to provide adequate perfusion despite what appears to be an adequate output to do so beautifully underscores the interaction between the systemic inflammatory response and the heart in sepsis patients [10].

The current management of sepsis-induced cardiomyopathy focuses largely on supportive care, targeting the restoration of systemic perfusion and blood pressure, as well as the treatment of the initiating infectious process [11]. From our perspective, the prompt initiation of vasopressor support and cautious fluid therapy, further supported by the initiation of targeted antimicrobial agents, plays a critical role in supporting cardiovascular stability for patients during the acute phase of sepsis. However, the long-standing consequences of septic cardiomyopathy, including the potential for myocardial recovery and residual functional disabilities, require further study outside the critical care environment [12]. After the acute phase, the patient was treated with a beta-blocker and an ACEI (angiotensin-converting enzyme inhibitor). The interventions are aimed at preventing cardiac remodelling and producing a long-term benefit on the cardiac status of survivors of septic shock. These therapeutic strategies are directed against the prolonged inflammation and oxidative stress processes that remain after an acute septic insult, mediating the protracted myocardial injury and remodelling [13].

The case reported highlights the necessity for prompt identification, along with aggressive treatment of septic cardiomyopathy, to avoid irreversible damage to the myocardium and ensure successful management of patients. This demands an improved understanding of the pathophysiology of this process in order to develop a more specific and effective therapeutic strategy, as opposed to traditional supportive care [14].

## Conclusions

Sepsis is one of the most commonly encountered presentations in the modern era of clinical medicine. Early recognition and prompt intervention form the cornerstones for the management of septic cardiomyopathy. This case manages to highlight that some of the inflammatory markers may still be in the normal range in the early stages of severe sepsis. Our case demonstrates that a conservative approach may be warranted for deep-seated infections, although this would vary from case to case. Lastly, collaborative multidisciplinary management is indispensable and improves outcomes in severe sepsis involving multi-organ failure.
